# New *Mesopsyllus* species from the Bohai Sea, China, re-evaluation of the validity of *Vibriopsyllus* Kornev & Chertoprud, 2008 and proposal of *Sympodella* gen. n. (Copepoda, Harpacticoida, Canthocamptidae)

**DOI:** 10.3897/zookeys.718.13700

**Published:** 2017-12-04

**Authors:** Fang-hong Mu, Rony Huys

**Affiliations:** 1 College of Marine Life Sciences, Ocean University of China, 5 Yushan Road, Qingdao 266003, China; 2 Department of Life Sciences, The Natural History Museum, Cromwell Road, London SW7 5BD, United Kingdom

**Keywords:** *Carolinicola*, Hemimesochrinae, *Isthmiocaris*, *Mesopsyllus dimorphus* sp. n., *M. spiniferus* sp. n., *Perucamptus*, *Pyrocletodes*, *Sympodella* gen. n.

## Abstract

Two new species of the genus *Mesopsyllus* Por, 1960 (Canthocamptidae) are described from the Bohai Sea, eastern China. *Mesopsyllus dimorphus* **sp. n.** and *M. spiniferus* **sp. n.** differ from their congeners by the presence of two instead of three outer spines on P2–P3 exp-3. They can be differentiated from each other by (1) number of inner setae on P3–P4 enp-2; (2) anterior margin of antennulary segment 7 of male; (3) ornamentation of male abdomen; (4) sexual dimorphism on P2 endopod and P3–P4 exp-3; and (5) differences in length of setae on male P5. Some observations in the original description of *M. atargatis* Por, 1960 are reinterpreted and the type material of *M. secundus* (Wells, 1965) is re-examined. Comparison between the type species of *Vibriopsyllus* Kornev & Chertoprud, 2008 and the four known species of *Mesopsyllus* shows the former as a junior subjective synonym of the latter. Consequently, *Vibriopsyllus curviseta* Kornev & Chertoprud, 2008 is formally transferred to *Mesopsyllus* as *M. curvisetus* (Kornev & Chertoprud, 2008), **comb. n.** A key to species and an updated generic diagnosis of *Mesopsyllus* are presented.

The taxonomic status of the genus *Carolinicola* Huys & Thistle, 1989 is re-evaluated. The characters of its type species, *C. trisetosa* (Coull, 1973), indicate that the latter (and – by inference – the genus *Carolinicola*) should remain in the Danielsseniinae. *Carolinicola galapagoensis* Mielke, 1997 is fixed as the type species of a new genus *Sympodella* **gen. n.** and placed in the Hemimesochrinae (Canthocamptidae) as the putative sistertaxon of *Pusillargillus* Huys & Thistle, 1989. The relationships and potential synonymy of the genera *Pyrocletodes* Coull, 1973, *Perucamptus* Huys & Thistle, 1989 and *Isthmiocaris* George & Schminke, 2003 are briefly discussed.

## Introduction

Sars (1920) proposed the monotypic genus *Hemimesochra* Sars, 1920 in the family Canthocamptidae for *H. clavularis* Sars, 1920. The species is extremely rare, being known from only five females collected from deepwater (91–101 m) muddy sediments off Risør, southern Norway (Sars 1920) and Loch Nevis, western Scotland (Wells 1965). Baguley (2004) recorded five specimens from the northern Gulf of Mexico deep sea which he assigned to Hemimesochra aff. clavularis. Monard (1927) continued to list *Hemimesochra* as a member of the Canthocamptidae in his *Synopsis universalis generum harpacticoidarum* while Lang (1936) transferred it to the family Cletodidae which he subdivided in a number of lineages (“Reihen”). *Hemimesochra* was initially placed in the *Heteropsyllus*-Reihe, together with *Heteropsyllus* T. Scott, 1894 and *Pontopolites* T. Scott, 1894. However, the Reihe concept was subsequently abandoned by Lang (1944, 1948).

Por (1960) established the genus *Mesopsyllus* Por, 1960 in the family Cletodidae for the type and only species, *M. atargatis* Por, 1960, from the Black Sea basin. In a later paper, Por (1964a) considered *Mesopsyllus* a junior subjective synonym of *Hemimesochra* in which was included a third, newly described, species, *H. derketo* Por, 1964a, from the Israeli Levantine coast. Lang (1965) rejected Por’s revised diagnosis of *Hemimesochra*, claiming the three species represented three monotypic genera and *H. derketo* (which he consistently misspelled as *dekerto*) should be placed in a new genus *Poria* Lang, 1965 (= *Hanikraia* Huys, 2009). Meanwhile and unbeknown to Lang (1965), Por (1964b) had further expanded the generic concept of *Hemimesochra* by adding two new species from the Swedish west coast, *H. nixe* Por, 1964b and *H. nympha* Por, 1964b, while Wells (1965) had described *H. secunda* Wells, 1965 from Loch Nevis. Additional, but radically divergent, species were subsequently included from the deep sea off North Carolina (*H. trisetosa* Coull, 1973a) and the Peru–Chile (Atacama) Trench (*H. rapiens* Becker, 1979) (Coull 1973a, Becker 1979). Finally, *Leimia dubia* Wells, 1965, originally described from the Scottish west coast, was transferred to *Hemimesochra* by Becker (1972, 1979), raising the number of species to seven.

Por (1986) removed the genera *Hemimesochra*, *Mesopsyllus* and *Poria* from the Cletodidae and placed them in a new subfamily Hemimesochrinae in the Canthocamptidae without making a proper recommendation for this course of action. Likewise, *Hemimesochra rapiens* was transferred as *species incertae sedis* to the Canthocamptidae without any justification. Huys and Thistle (1989) reviewed the relationships within the heterogeneous genus *Hemimesochra* and redistributed the seven species over six genera, four of which proposed as new. Following this revision, *Hemimesochra* remained monotypic with *H. clavularis* as its only member. *Hemimesochra secunda* was transferred as *M. secundus* to the genus *Mesopsyllus* while the new genera *Boreolimella* Huys & Thistle, 1989 (*H. dubia*, *H. nympha*), *Carolinicola* Huys & Thistle, 1989 (*H. trisetosa*), *Perucamptus* Huys & Thistle, 1989 (*H. rapiens*) and *Pusillargillus* Huys & Thistle, 1989 (*H. nixe*) accommodated the remaining species (Huys and Thistle 1989).

Karaytuğ and Huys (2004) recognized within the primarily freshwater Canthocamptidae a core complex of genera confined to marine and brackish water habitats which they called the *Mesochra*-group. This group, which is fundamentally different from Por’s (1986) taxonomic concept of the Hemimesochrinae, comprises the genera *Mesochra* Boeck, 1865, *Parepactophanes* Kunz, 1935, *Mesopsyllus*, *Psammocamptus* Mielke, 1975, *Taurocletodes* Kunz, 1975, *Amphibiperita* Fiers & Rutledge, 1990, *Bathycamptus* Huys & Thistle, 1989, and *Isthmiocaris* George & Schminke, 2003. Members of this group share the reduced morphology of the male sixth legs (unconfirmed in *Parepactophanes*), being represented by membranous flaps completely lacking in armature elements. In the females the sixth legs closing off the genital apertures bear 1–3 setae, indicating a different ontogenetic trajectory between the sexes. The genera *Hemimesochra*, *Poria*, *Boreolimella*, *Perucamptus* and *Pusillargillus* which are known from females only, were also regarded as representatives of the *Mesochra*-group based on their close similarity in mouthpart morphology with *Bathycamptus*, *Mesopsyllus* and *Psammocamptus*. The current understanding of relationships within this group is insufficient since many species are incompletely described or known from only one sex. The discovery of two new species of *Mesopsyllus* from the Bohai Sea (one of which was cited as *Mesopsyllus* sp. 2 in Mu et al. (2001)) provides us with an opportunity to update its generic diagnosis, including new information about the male. In this paper we present descriptions of both species, provide a key to species of *Mesopsyllus*, assess the validity of a recently established genus, *Vibriopsyllus* Kornev & Chertoprud, 2008, from the White Sea, and re-evaluate the taxonomic position of *Carolinicola*.

## Materials and methods

**Figure 1.**
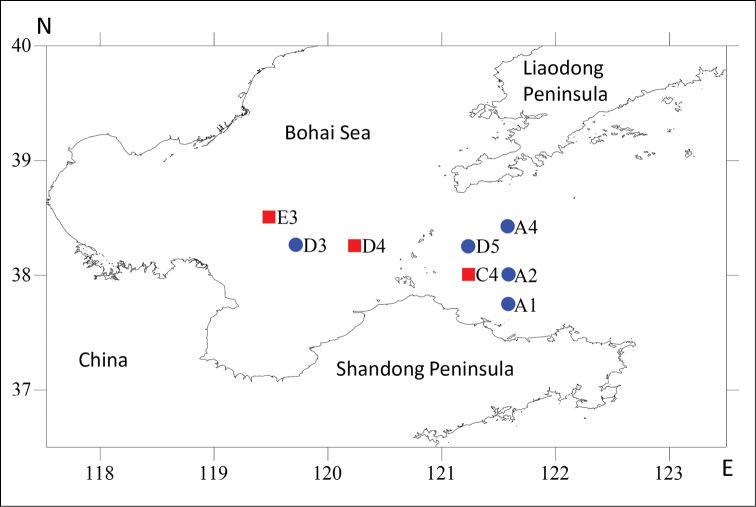
Location of sampling stations in the Bohai Sea, China where *Mesopsyllus spiniferus* sp. n. and *M. dimorphus* sp. n. were observed, indicated by red squares and blue circles, respectively.

Specimens were collected during 1998–1999 from the central region and the strait of the Bohai Sea (Fig. 1) in eastern China. Sediments ranged from muddy sand to pure mud. Samples were collected with a 0.1 m^2^ box corer at an average depth of 20 m (range 11–70 m). Standard subsamples were taken from the box corer by three 26 mm diameter plastic tubes inserted to a depth of 5 cm and were subsequently fixed in 10% formalin. Meiofauna was extracted by Ludox centrifugation flotation. Harpacticoids were sorted and preserved in 4% formalin. Prior to dissection the habitus was drawn from whole specimens temporarily mounted in lactophenol. Specimens were dissected in lactic acid and the parts individually mounted in lactophenol under coverslips which were subsequently sealed with transparent nail varnish.

All drawings were prepared using a camera lucida on a Zeiss Axioskop differential interference contrast microscope. The terminology for body and appendage morphology follows that of Huys and Boxshall (1991) and Huys et al. (1996). Abbreviations used in the text and Tables 2 and 4 are A1 for antennule, A2 for antenna, P1–P6 for thoracopods 1–6, exp for exopod, enp for endopod, benp for baseoendopod, exp(enp)-1(2, 3) to denote the proximal (middle, distal) segment of a ramus; apo for apohysis, and ae for aesthetasc. The setae on P5 are counted from the innermost on each ramus (as in P1–P4). Body length was measured from the anterior margin of the cephalic shield to the posterior margin of the anal somite. Scale bars in all illustrations are in µm. Type material is deposited in The Natural History Museum, London (NHMUK).

## Systematics

### Order Harpacticoida Sars, 1903

#### Family Canthocamptidae Brady, 1880

##### Subfamily Hemimesochrinae Por, 1986

###### Genus Mesopsyllus

Taxon classificationAnimaliaHarpacticoidaCanthocamptidae

Por, 1960

####### Diagnosis.

Rostrum not defined at base; triangular. Antennule 6-segmented in ♀, with aesthetasc on segments 4 and 6; 8-segmented, haplocer with geniculation between segments 6 and 7 in ♂; with enlarged modified spines on segments 2–3 and 6 in ♀, and segments 2–4 in ♂. Antenna with one abexopodal seta on allobasis; exopod 1-segmented, with 2–3 setae. Mandibular palp with short basis (with one seta), 1-segmented endopod (with four setae) and vestigial unisetose exopod. Maxillule with rami incorporated into basis. Maxilla with two endites on syncoxa; endopod discrete. Maxilliped with well developed seta on syncoxa. Swimming legs of ♀ with 3-segmented exopods and 3- (typical condition in P1) or 2-segmented endopods (P2–P4, unusual condition in P1). Setal formulae of P1–P4 as follows:

**Table:** 

**Thoracopod**	**Exopod**	**Endopod**
P1	0.1.022	1.1.111 or 1.111
P2	0.1.12[2–3]	1.[1–2]21
P3	0.1.22[2–3]	1.[1–2]21 (♀) or 1.1+apo.020 (♂)
P4	0.1.[1–2]2[2–3]	1.[1–2]21

Inner seta of P1 enp-1 short, not recurved backwardly and dorsally; outer spine of P1 exp-1 not enlarged; outer exopodal spines of P1–P4 sparsely bipinnate, in P2–P4 without elongate pinnules in proximal half. P2 endopod occasionally with sexual dimorphism (inner seta of enp-1 distinctly shorter in ♀; enp-2 with additional inner seta in ♀). P3 endopod 3-segmented in ♂; enp-2 with inner seta and slender terminal apophysis; enp-3 with two apical setae. P4 enp-1 and sometimes enp-2 and exp-3 with slight sexual dimorphism (setal lengths). P4 exp-3 occasionally sexually dimorphic (length of proximal inner seta). P5 with discrete exopod and baseoendopod; exopod small, with 3–5 and 4–5 elements in ♀ and ♂, respectively; endopodal lobe with four and two elements in ♀ and ♂, respectively. Sixth pair of legs asymmetrical in ♂, unarmed. Caudal ramus short, with six setae.

####### Type species.

*Mesopsyllus atargatis* Por, 1960 (by monotypy).

####### Other species.

*Mesopsyllus secundus* (Wells, 1965), *M. curvisetus* (Kornev & Chertoprud, 2008), comb. n., *M. dimorphus* sp. n., *M. spiniferus* sp. n.

###### Mesopsyllus dimorphus sp. n.

Taxon classificationAnimaliaHarpacticoidaCanthocamptidae

http://zoobank.org/EF350CF9-712C-4BD0-8301-1134C452CBD6

**Figure 2.**
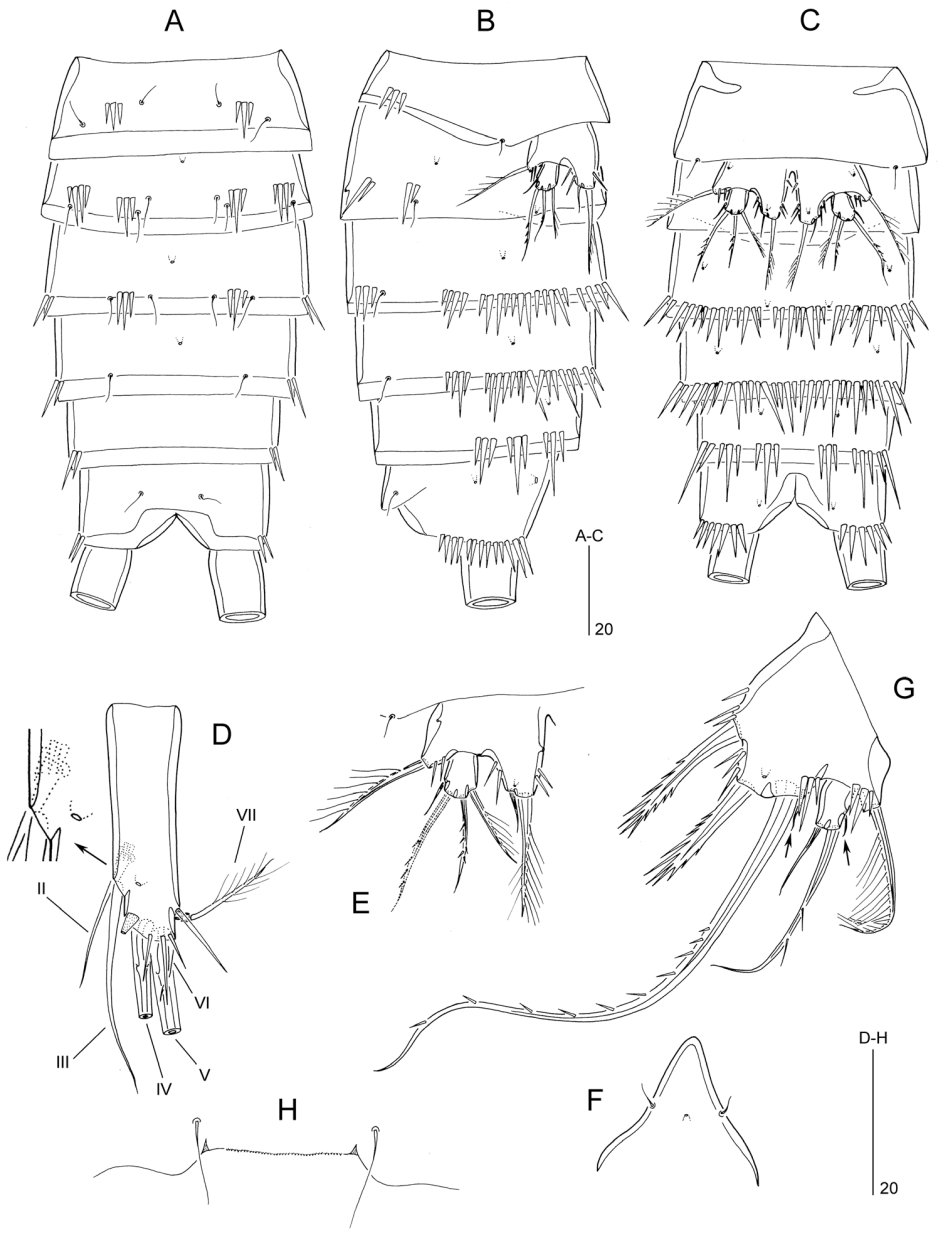
*Mesopsyllus dimorphus* sp. n.: **A** urosome ♂, dorsal **B** urosome ♂, lateral **C** urosome ♂, ventral **D** caudal ramus ♂, ventral (inset showing spinules around base of seta II) **E** P5 ♂, anterior **F** rostrum ♂, dorsal **G** P5 ♀, anterior (minute setae on exopod and endopodal lobe indicated by arrow) **H** anal operculum ♀. [Caudal rami in A–C not drawn at full length]. **A–F** based on holotype (NHMUK reg. no. 2013.1033), **G–H** based on paratype (NHMUK reg. no. 2013.1034).

**Figure 3.**
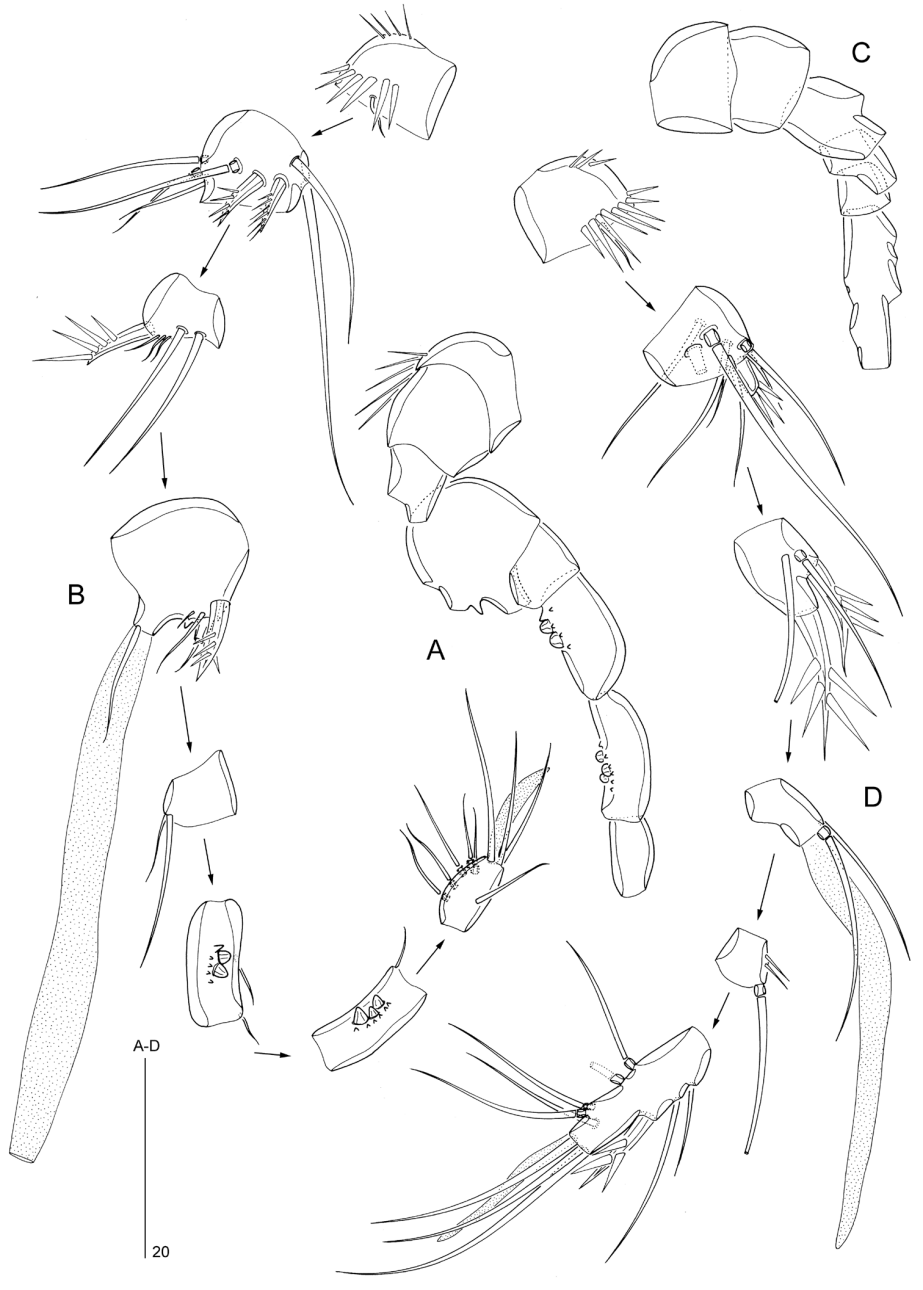
*Mesopsyllus dimorphus* sp. n.: **A** antennule ♂ (armature omitted), ventral **B** antennule ♂ (disarticulated), ventral **C** antennule ♀ (armature omitted), ventral **D** antennule ♀ (disarticulated), ventral **A–B** based on holotype (NHMUK reg. no. 2013.1033) **C–D** based on paratype (NHMUK reg. no. 2013.1034).

**Figure 4.**
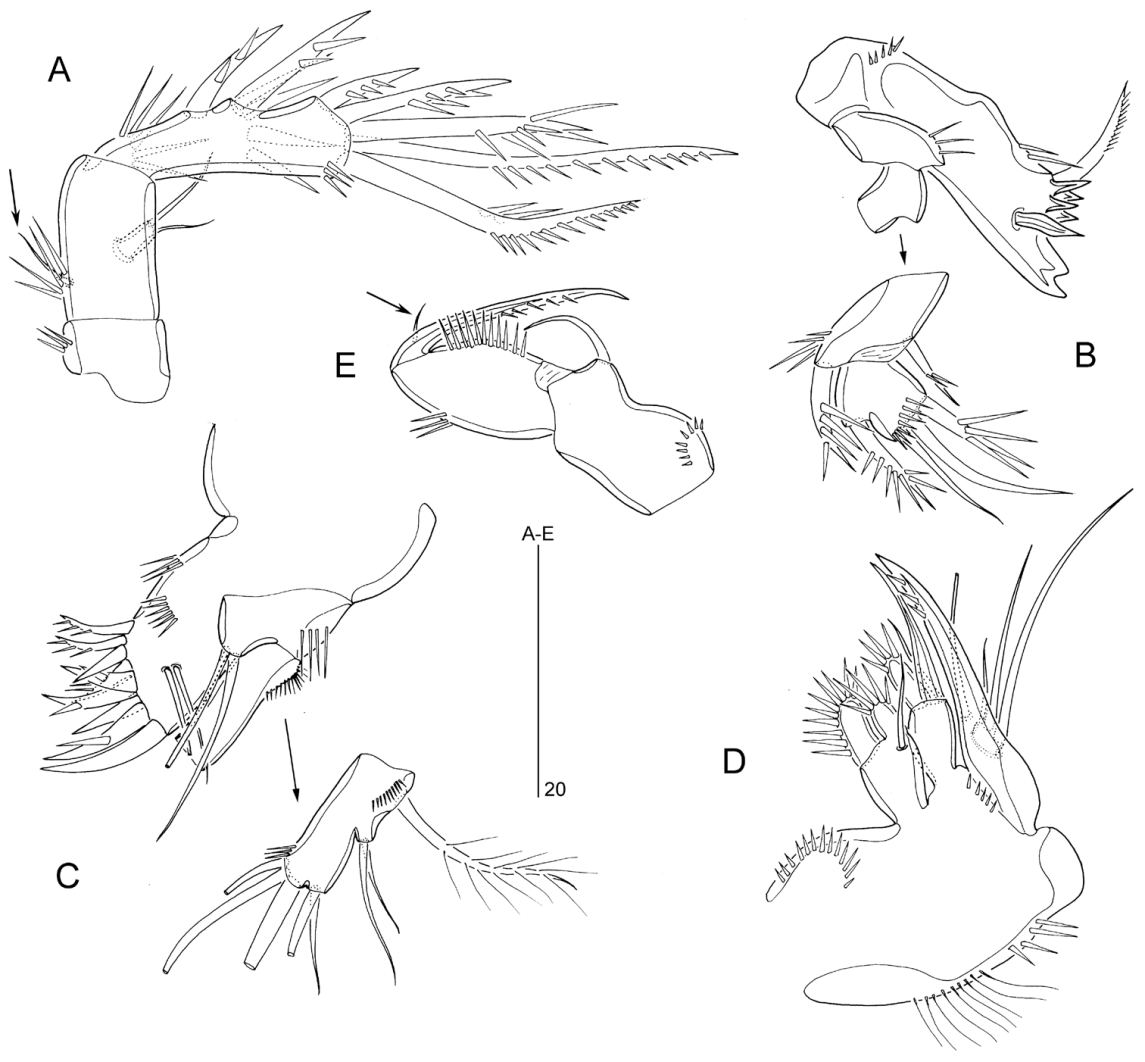
*Mesopsyllus dimorphus* sp. n. (♂): **A** antenna (abexopodal seta on allobasis indicated by arrow) **B** mandible (with palp disarticulated) **C** maxillule (with palp disarticulated) **D** maxilla **E** maxilliped (vestigial seta on endopod indicated by arrow). All drawings based on holotype (NHMUK reg. no. 2013.1033).

**Figure 5.**
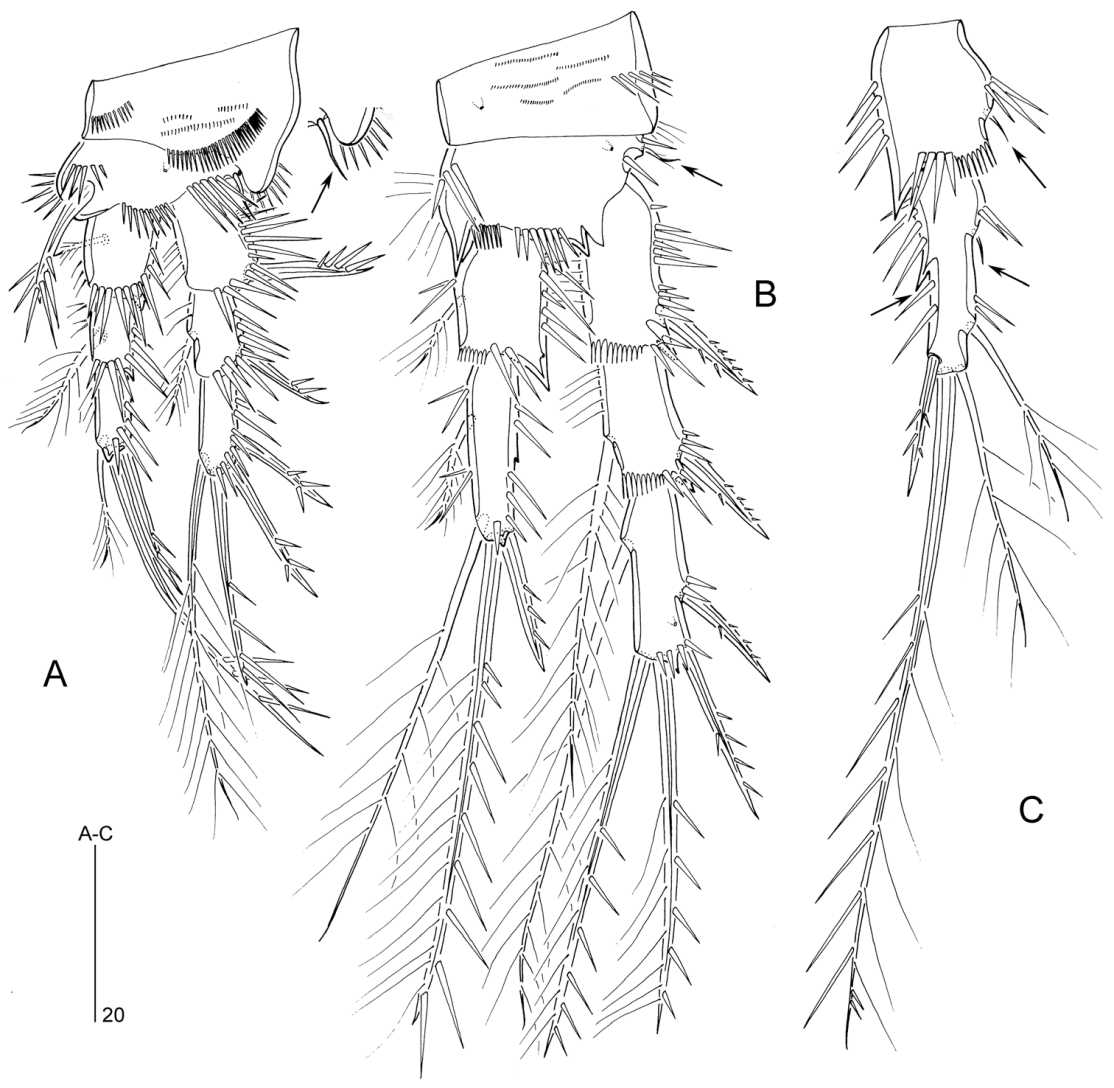
*Mesopsyllus dimorphus* sp. n.: **A** P1 ♂, anterior (outer basal seta indicated by arrow in inset) **B** P2 ♂, anterior (outer basal seta indicated by arrow) **C** P2 endopod ♀, anterior (vestigial setae along inner margin and hook-like process along outer margin indicated by arrows) **A–B** based on holotype (NHMUK reg. no. 2013.1033), **C** based on paratype (NHMUK reg. no. 2013.1034).

**Figure 6.**
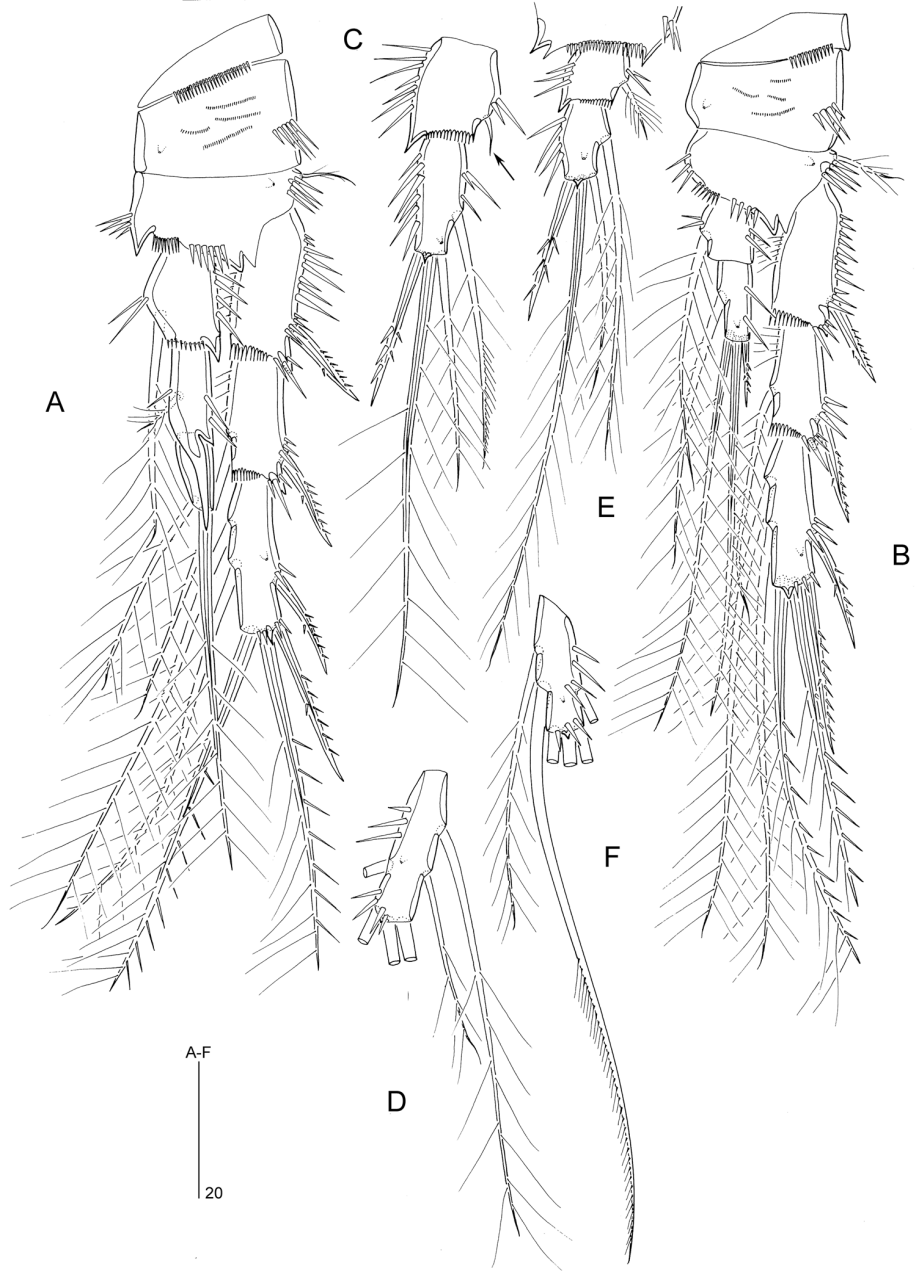
*Mesopsyllus dimorphus* sp. n.: **A** P3 ♂, anterior **B** P4 ♂, anterior **C** P3 endopod ♀, anterior (vestigial seta indicated by arrow) **D** P3 exp-3 ♀ (apical and outer elements not drawn at full length), anterior **E** P4 endopod and distal portion of basis ♀, anterior **F** P4 exp-3 ♀ (apical and outer elements not drawn at full length), anterior **A–B** based on holotype (NHMUK reg. no. 2013.1033) **C–F** based on paratype (NHMUK reg. no. 2013.1034).

**Figure 7.**
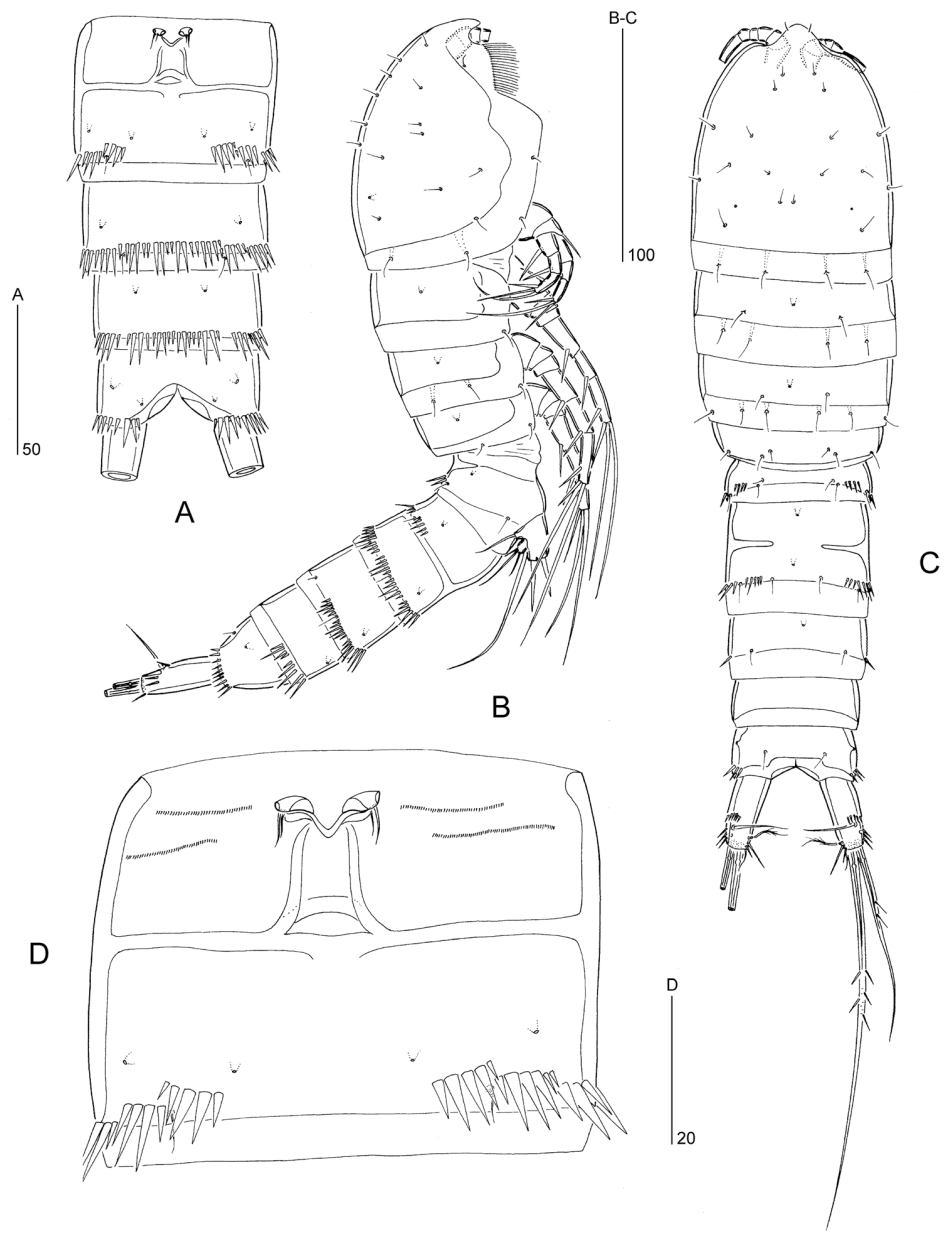
*Mesopsyllus dimorphus* sp. n. (♀): **A** urosome (excluding P5-bearing somite; distal portion of caudal rami omitted), ventral **B** habitus, lateral **C** habitus, dorsal **D** genital double-somite, ventral (copulatory pore indicated by arrow). All drawings based on paratype (NHMUK reg. no. 2013.1034).

Figures 2 , 3 , 4 , 5 , 6 , 7

####### Type locality.

Eastern China, Strait of the Bohai Sea, sampling locality D5 (38°15'N, 121°15'E); 37.0 m depth; very fine sand (Fig. 1; Table 1).

**Material examined.** Holotype: adult ♂ dissected on 13 slides (NHMUK reg. no. 2013.1033). Paratypes are 1 ♀ dissected on 17 slides (NHMUK reg. no. 2013.1034), and 10 ♀♀ and 2 ♂♂ preserved in ethanol (NHMUK reg. nos 2013.1035–1044); all paratypes were collected from the type locality. Additional material was collected from stations A1 (37°44'N, 121°35'E), A2 (38°N, 121°35'E), A4 (38°25'N, 121°35'E) and D3 (38°15'N, 119°44'E) in the central part and the strait of the Bohai Sea, eastern China (Fig. 1; Table 1). Collected by F.-h. Mu and Y.-q. Guo in September 1998.

**Table 1.** Location and environmental characteristics of sampling stations in the Bohai Sea (Md = median grain size).

Station	Lattitude / Longitude	Depth (m)	Sediment type	Md
A1	37°44'N, 121°35'E	20.5	coarse silt	5.42
A2	38°00'N, 121°35'E	42.8	sandy silt	5.4
A4	38°25'N, 121°35'E	50.8	very fine sand	3.87
C4	38°00'N, 121°15'E	23.8	sandy silt	5.19
D3	38°15'N, 119°44'E	22.9	coarse silt	5.73
D4	38°15'N, 120°15'E	24.3	silty sand	4.96
D5	38°15'N, 121°15'E	37.0	very fine sand	3.94
E3	38°30'N, 119°30'E	26.0	clayey silt	7.63

####### Description of male.

Body length 220–280 µm (n = 3, mean = 250 µm). Body slightly tapering posteriorly as in ♀ (compare Fig. 7B–C). P1-bearing somite fused with cephalothorax. Pleural margins of cephalic shield furnished with long hair-like setules (as shown for female in Fig. 7B). Body covered with pattern of minute pimples (not figured). Hyaline frills plain (as shown for female in Fig. 7B–C). Posterior margin of anal operculum straight and with fine denticles (as figured for ♀ in Fig. 2H); anus terminal.

Body ornamentation (Fig. 2A–C). All somites with integumental sensilla, except for penultimate one. Pores present on all somites (positions on urosomites indicated in Fig. 2A–C). Prosome without spinular ornamentation. Pattern of spinular rows on urosome as follows: urosomite-1 with short paired dorsal rows; urosomite-2 with pairs of short rows dorsally and dorsolaterally; urosomite-3 with a pair of short rows dorsally and a long, continuous row stretching ventrally and laterally; urosomite-4 without dorsal spinules but with a long, continuous row ventrally and laterally; urosomite-5 without dorsal spinules but with an interrupted row ventrally and laterally; anal somite with lateral and ventral spinules at base of caudal rami.

Rostrum (Fig. 2F) not defined at base; triangular with a round apex; with a pair of lateral sensilla subapically and a median pore dorsally.

Antennule (Fig. 3A–B) 8-segmented, haplocer with geniculation located between segments 6 and 7. Segment 1 with spinules along anterior and subdistal margins and one minute seta. Segment 2 with two stout spinulose spines and six smooth setae. Segment 3 with one stout spinulose spine, two long and three minute, naked setae. Segment 4 moderately swollen, with one stout pinnate spine, three short, naked setae (one of which arising from minute cylindrical process) and one small spiniform process near anterior distal corner; ventral surface of segment 4 with a sub-cylindrical setophore carrying one slender seta and one large aesthetasc. Segment 5 with two naked setae. Segment 6 with two slender setae and two conical elements (modified setae). Segment 7 with three conical elements and one anterodistal seta. Segment 8 with seven naked setae and apical acrothek consisting of two setae and short aesthetasc.

Antenna (Fig. 4A). Coxa well developed, bearing row of spinules. Allobasis without trace of original segmentation; with row of spinules and short smooth seta in proximal half of abexopodal margin. Exopod 1-segmented, about twice as long as wide; with two apical naked setae. Free endopod 1-segmented, bearing two surface rows of stout spinules and two stout pinnate spines along lateral margin; apical armature consisting of five pinnate spines; outer distal corner with few spinules.

Mandible (Fig. 4B). Gnathobase with strong teeth and unipinnate seta at dorsal corner, with spinular ornamentation as illustrated. Palp consisting of basis, 1-segmented endopod and vestigial exopod. Basis short, with few spinules and strong pinnate spine near inner distal corner. Endopod with one pinnate inner spine; apical margin with one pinnate spine, two naked setae and transverse spinular row. Exopod represented by a short pinnate spine.

Maxillule (Fig. 4C). Praecoxa with well-developed arthrite bearing two setae and two spinular rows on anterior surface, and nine spines along distal margin. Coxa with long spinules along outer margin; with cylindrical endite bearing two apical setae. Basis and rami fused, forming elongate palp; with spinules on inner and outer margins as figured; basal armature presumably consisting of five naked setae; endopod represented by small cylindrical outgrowth with two distal setae; exopod represented by one long plumose seta.

Maxilla (Fig. 4D). Syncoxa with three rows of spinules, a row of setules and two (coxal) endites; proximal endite with a fused spinulose process, one spinulose spine and one short naked seta; distal endite with one spinulose spine, one naked spine and one naked seta. Allobasis drawn into slightly curved claw, bearing few spinules near apex and naked seta halfway down the claw. Exopod a minute segment with three naked setae.

Maxilliped (Fig. 4E) subchelate. Syncoxa with row of small spinules near base and naked seta at inner distal corner. Basis with spinular row along most of palmar (inner) margin and few spinules halfway along outer margin; unarmed. Endopod represented by a strong, acutely curved claw, spinulose along inner distal half and with one minute seta near base.

Swimming legs with 3-segmented exopods and 3- (P1, P3) or 2-segmented endopods (P2, P4).

P1 (Fig. 5A). Praecoxa (not illustrated) a well-developed U-shaped. Coxa with two rows of long spinules and additional small spinules on anterior surface as figured; outer distal corner produced into a round bulge, bearing spinules posteriorly. Basis bearing short outer seta (indicated by arrow in inset of Fig. 5A) and stout bipinnate inner spine; anterior surface with pore and three spinule rows; additional spinules along inner margin. Exopodal and endopodal segments with spinules along outer and distal margins, and with sparse setules along inner margin (except exp-3). Exp-1 with pinnate outer spine; exp-2 with plumose inner seta and pinnate outer spine; exp-3 with two outer and one apical pinnate spines, and one subdistal plumose seta. Enp-1 and enp-2 each with plumose inner seta and small spinous process at outer distal corner; enp-3 with plumose inner seta subdistally and two pinnate elements apically.

P2 (Fig. 5B). Praecoxa (not illustrated) a well-developed U-shaped sclerite with spinules along its distal margin. Coxa with a row of long spinules along outer margin and few long setules near outer distal corner; anterior surface with a pore and rows of tiny spinules as figured. Basis with short outer seta; with spinules along inner, distal and outer margins; inner margin also with hair-like setules; with pore on anterior surface; inner distal corner produced into sharp spinous process; distal margin between exopod and endopod with spinous process. Exopodal segments with spinules along outer margin; exp-1 and -2 with setules along inner margin and spinous process at outer distal corner; exp-2 with plumose inner seta and pinnate outer spine; exp-3 with one plumose inner seta, two pinnate spines (with setules on inner and spinules on outer margin) distally and two pinnate outer spines; exp-3 with pore on anterior surface and spinules along distal margin. Endopodal segments with spinules along outer, inner and distal margins; enp-1 with short, plumose inner seta and spinous process at outer distal corner; enp-2 with one short, plumose inner seta, one plumose seta subdistally, one long pinnate spiniform seta distally and one unipinnate outer spine; outer margin of enp-2 with small spike halfway down the segment length, possibly indicating ancestral segmentation boundary. Intersegmental hyaline frills of segments well developed, serrate.

P3 (Fig. 6A). Praecoxa a well-developed U-shaped sclerite with spinules along its distal margin. Coxa and basis as in P2, except for presence of setules along inner margin of basis. Proximal and middle exopodal segments as in P2; distal segment with two plumose inner setae, two pinnate spines apically and two pinnate outer spines. Endopod 3-segmented; enp-1 and -2 with spinules along outer and inner margins; enp-1 with long, plumose inner seta, a row of short spinules along distal margin, and a spinous process at outer distal corner; enp-2 with one short, plumose inner seta; distal margin of enp-2 with outer spinous process and long anterior apophysis extending beyond enp-3; enp-3 (pseudosegment originating from secondary subdivision of ancestral enp-2) with two plumose setae apically.

P4 (Fig. 6B). Praecoxa, coxa and basis as in P2–P3, except spinous process at inner distal corner of basis absent; inner margin of basis without setular ornamentation. Exopod as in P3. Endopod short, reaching just beyond distal margin of exp-1; enp-1 with plumose inner seta and few spinules along outer and inner margins; enp-2 with one plumose inner seta, two plumose setae apically and one short, pinnate outer spine; with sparse spinular ornamentation along outer margin and pore on anterior surface.

P5 (Fig. 2E). Baseoendopods of fifth pair of legs fused medially forming deeply incised plate. Baseoendopod and exopod not fused, the former with outer basal seta. Endopodal lobe conical, reaching to apical margin of exopod; with one plumose inner spine and one short, naked outer seta; with sparse spinules along outer and inner margins. Exopod small, slightly longer than wide; with four elements: one plumose inner seta, two pinnate apical setae and one small, naked outer seta.

P6 (Fig. 2C). Fused to genital somite; represented by a median lobe without armature.

Caudal ramus (Fig. 2D). About 3.1 times as long as maximum width; with long spinules around insertion sites of setae IV–VII and short spinules around base of seta II. Ventral surface with pore near seta III and tube-pore near distal outer corner. Armature consisting of six setae (seta I apparently absent); setae II–III slender and bare, positioned along distal half of outer margin; seta IV–V well developed, pinnate; seta V about twice as long as seta VI and about half as long as the body length; seta VI small and naked; seta VII tri-articulated at base, laterally displaced and inserting near distal inner corner.

####### Description of female.

Body length 240–330 µm (n = 10, mean = 292 µm). General body shape (Fig. 7B–C) as in male. Body covered with pattern of minute pimples (not figured). Sexual dimorphism in antennule, P2–P6, and urosomal segmentation and ornamentation.

Urosome (Fig. 7A–C). Genital and first abdominal somites fused forming genital double-somite; original segmentation marked by internal, transverse chitinous ribs laterodorsally, laterally and ventrally. Spinular ornamentation as follows: urosomite-1 with short paired dorsolateral rows; genital double-somite with short, paired lateral rows in anterior half (urosomite-2) and paired lateral rows extending laterodorsally and lateroventrally in posterior half (urosomite-3); urosomite-4 and urosomite-5 each with a long row, extending ventrally and ventrolaterally; anal somite with lateral and ventral rows around bases of caudal rami. Gonopores (Fig. 7D) fused, forming common median genital slit. P6 represented by two minute setae. Copulatory pore large, located in centre of genital double-somite; anterior half of genital double-somite with paired rows of minute spinules either side of genital slit.

Antennule (Fig. 3C–D) short, 6-segmented. Segment 1 with two spinule rows and one minute seta; segment 2 with eight naked setae (two with bi-articulated base) and one spinulose spine; segment 3 with two stout spinulose spines and two slender setae (one with bi-articulated base); segment 4 with large aesthetasc fused basally to short seta and one slender bi-articulated seta; segment 5 with few spinules and one bi-articulated naked seta along anterior margin; distal segment with one stout spinulose spine, nine naked setae (four bi-articulated at base) and apical acrothek consisting of slender seta and short aesthetasc.

P2 (Fig. 5C). Coxa, basis and exopod as in ♂. Endopod 2-segmented. Enp-1 with one minute inner seta (indicated by arrow) and spinules along outer, distal and inner margins; outer distal corner produced into spinous process. Enp-2 with spinules along inner and outer margins and a sharp hook halfway along outer margin (indicating ancestral segmentation); inner margin with two setae, proximal one (homologue of inner seta in ♂) minute (indicated by arrow); armature around distal margin as in ♂ except for plumose inner distal seta distinctly shorter.

P3 (Fig. 6C–D). Coxa, basis and first two segments of exopod as in ♂. Exp-3 with two plumose inner setae as in ♂ but distal one markedly shorter. Endopod 2-segmented, with spinules along inner and outer margins of both segments. Enp-1 with minute inner seta (indicated by arrow) and outer distal corner produced into spinous process. Enp-2 with one plumose inner seta, two plumose distal setae and one pinnate outer spine; distal margin with small spinous process; anterior surface with pore.

P4 (Fig. 6E–F). Coxa and first two segments of exopod as in ♂. Basis inner distal corner and distal margin between exopod and endopod with spinous process. Exp-3 (Fig. 6F) with two inner setae; proximal one considerably shorter than in ♂, distal one long and thick, unipinnate (rather than plumose as in ♂) in distal half. Endopod (Fig. 6E) 2-segmented, with spinules along outer margin of both segments and inner margin of enp-1. Enp-1 with small, plumose inner seta and spinous process at outer distal corner. Enp-2 with one plumose inner seta, two plumose distal setae and one pinnate outer spine, the latter much longer than in ♂; anterior surface with pore.

Seta and spine formulae of P1–P4 as follows:

**Table:** 

**Thoracopod**	**Exopod**	**Endopod**
P1	0.1.022	1.1.111
P2	0.1.122	1.121 (1.221)
P3	0.1.222	1.1+apo.020 (1.121)
P4	0.1.222	1.121

Formulae in parentheses denote female condition.

P5 (Fig. 2G). Fifth pair of legs not fused medially. Baseoendopod and exopod discrete. Endopodal lobe trapezoid with stepped distal margin; with spinules as figured and pore on anterior surface; armature consisting of four elements: innermost two spiniform, serrate and subequal in length, 3^rd^ one very long, pinnate and typically bent medially, and innermost one (indicated by arrow) minute, naked and setiform. Exopod small, longer than wide; with three setae: outermost one (indicated by arrow) minute and naked, middle one longest and unipinnate, and outermost short and naked.

####### Variability.

One female specimen shows an asymmetrical armature pattern on P4 exp-3, having one inner seta on one side and two on the other.

####### Etymology.

The species name is derived from the Greek *dis*, meaning twice, and *morphe*, meaning form, and alludes to the sexual dimorphism on P2–P4.

###### Mesopsyllus spiniferus sp. n.

Taxon classificationAnimaliaHarpacticoidaCanthocamptidae

http://zoobank.org/CE004F59-6DAC-4B88-B63D-C3A524A05AFB

Figures 8 , 9 , 10

####### Type locality.

Eastern China, strait of Bohai Sea, sampling locality C4 (38°00'N, 121°15'E); 23.8 m depth; sandy silt (Fig. 1; Table 1).

####### Material examined.

Holotype: adult ♂ dissected on 16 slides (NHMUK reg. no. 2013.1045). Paratypes are 2 ♀♀ dissected on 15 and 17 slides, respectively (NHMUK reg. nos 2013.1046–1047), and 1 ♂ preserved in alcohol (NHMUK reg. no. 2013.1048); one paratype collected from type locality, others from the central Bohai Sea, localities D4 (38°15'N, 120°15'E) and E3 (38°30'N, 119°30'E), respectively (Fig. 1; Table 1). Collected by F.-h. Mu and Y.-q. Guo in September 1998 and April 1999.

Since the new species is very similar to *M. dimorphus* its description is largely restricted to those features which are different.

####### Description of male.

**Figure 8.**
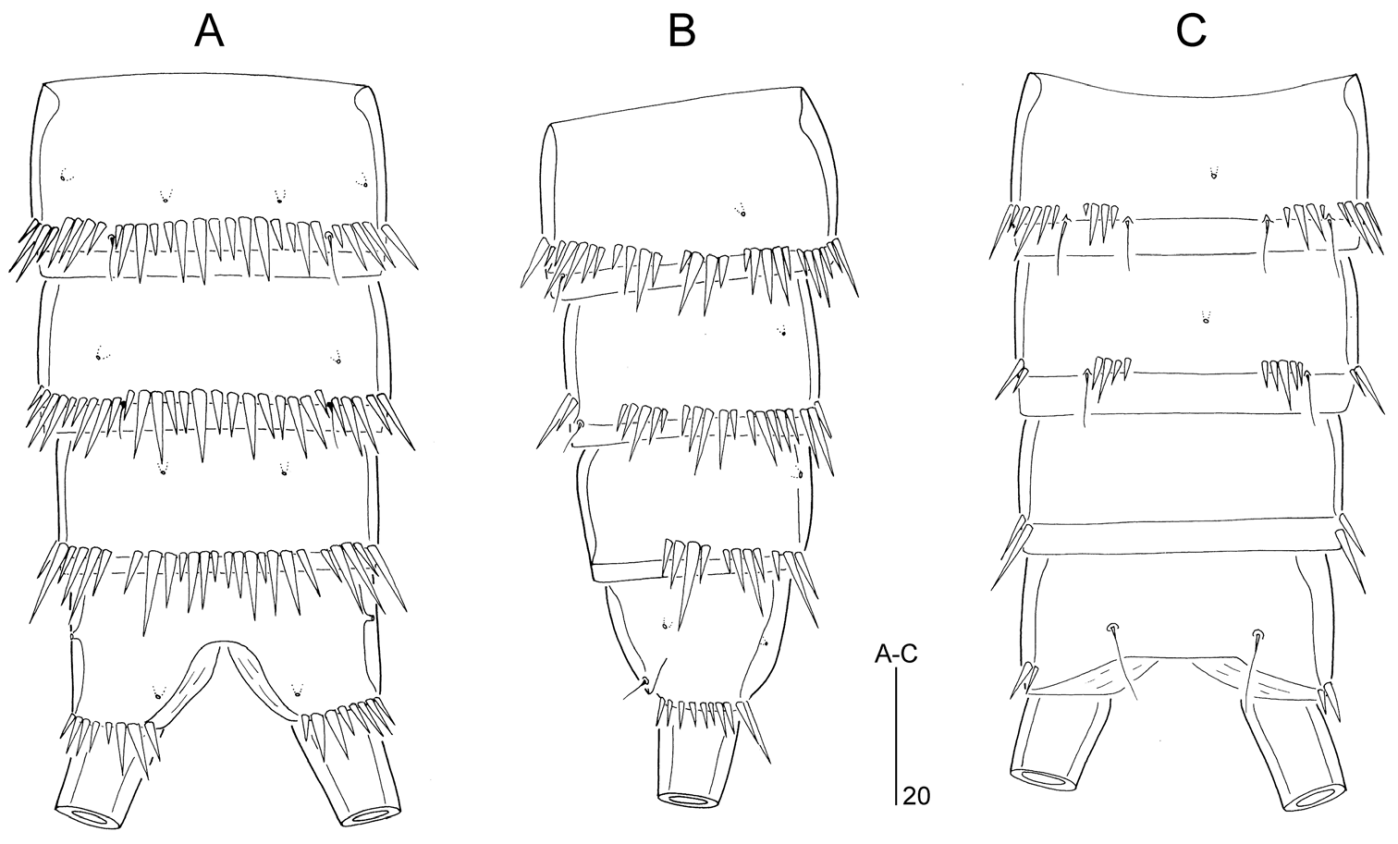
*Mesopsyllus spiniferus* sp. n. (♂): **A** abdomen, ventral **B** abdomen, lateral **C** abdomen, dorsal [Caudal rami in **A–C** not drawn at full length]. All drawings based on holotype (NHMUK reg. no. 2013.1045).

Body length 280–320 µm (n = 2, mean = 300 µm). Body covered with pattern of minute pimples (not figured). Urosomal ornamentation (Fig. 8A–C) very similar to that of *M. dimorphus* except for presence of one additional pair of dorsal spinule rows on urosomite-4.

Antennae, mouthparts, P6, caudal rami and rostrum as in *M. dimorphus*.

**Figure 9.**
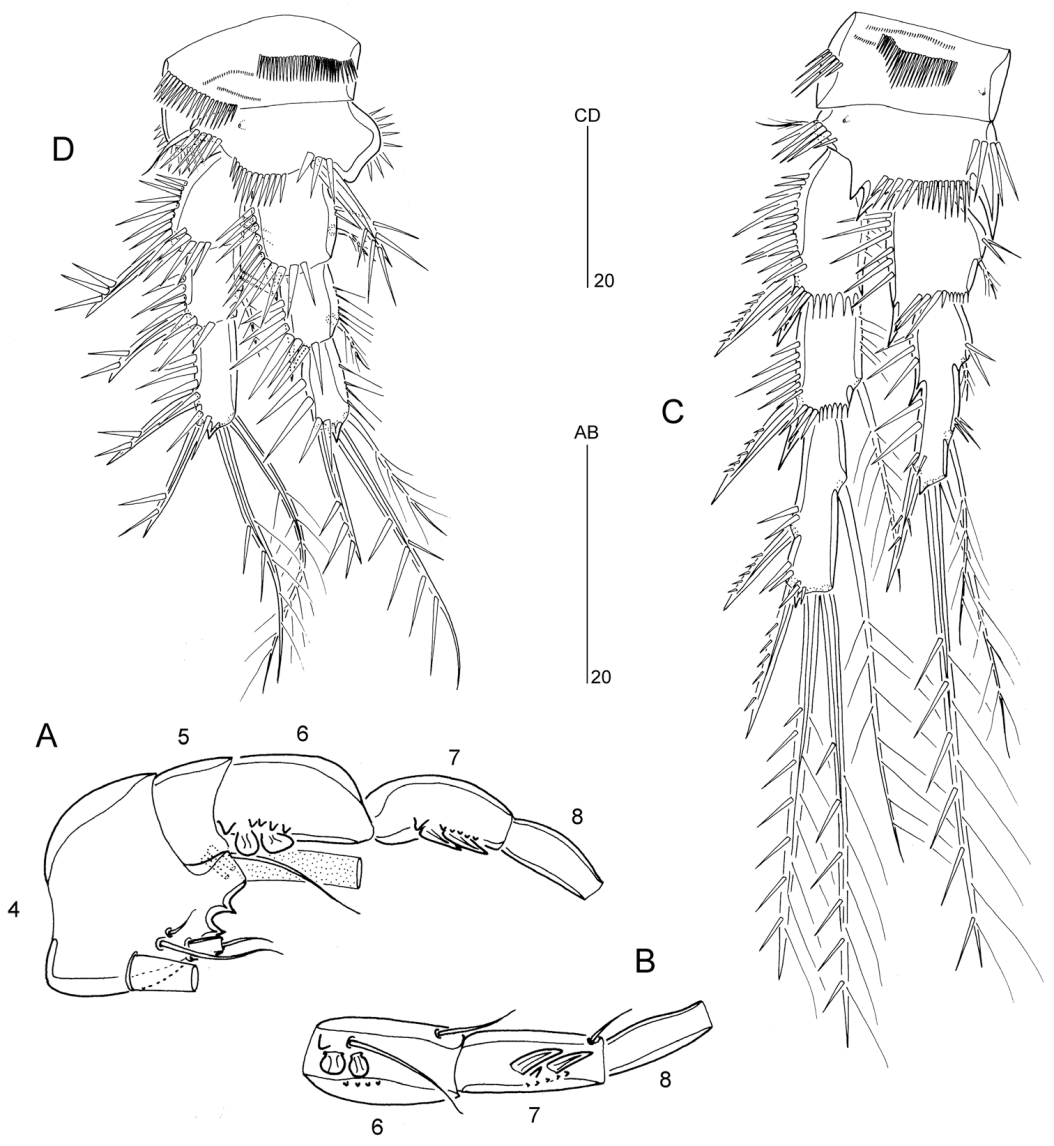
*Mesopsyllus spiniferus* sp. n.: **A** antennule ♂, distal five segments, showing modified elements on segments 6–7 (armature elements on other segments omitted or not drawn at full length), ventral **B** antennule ♂, distal three segments, showing modified elements on segments 6–7 (elements on segment 8 omitted), anterior **C** P2 ♂, anterior **D** P1 ♀, anterior **A–C** based on holotype (NHMUK reg. no. 2013.1045), **D** based on paratype (NHMUK reg. no. 2013.1046).

Antennule (Fig. 9A–B) 8-segmented. Anterior margin of segment 7 with two spiniform elements (modified setae) instead of three conical elements in *M. dimorphus*.

P1 with different spinular ornamentation on coxa, as figured for ♀ (Fig. 9D). Endopod shorter and inner seta on enp-2 distinctly shorter than in *M. dimorphus*.

P2 (Fig. 9C). Coxa with a row of long spinules on anterior surface. Inner seta of exp-2 shorter than in *M. dimorphus*, only extending to distal margin of exp-3. Endopod 2-segmented. Enp-1 with 1 minute inner seta and spinules along outer, inner and distal margins; outer distal corner produced into spinous process. Enp-2 with spinules along inner and outer margins and a sharp spinous process halfway down outer margin; inner margin with two setae, proximal of which minute and plumose; distal margin with two apical setae and one outer spine.

**Figure 10.**
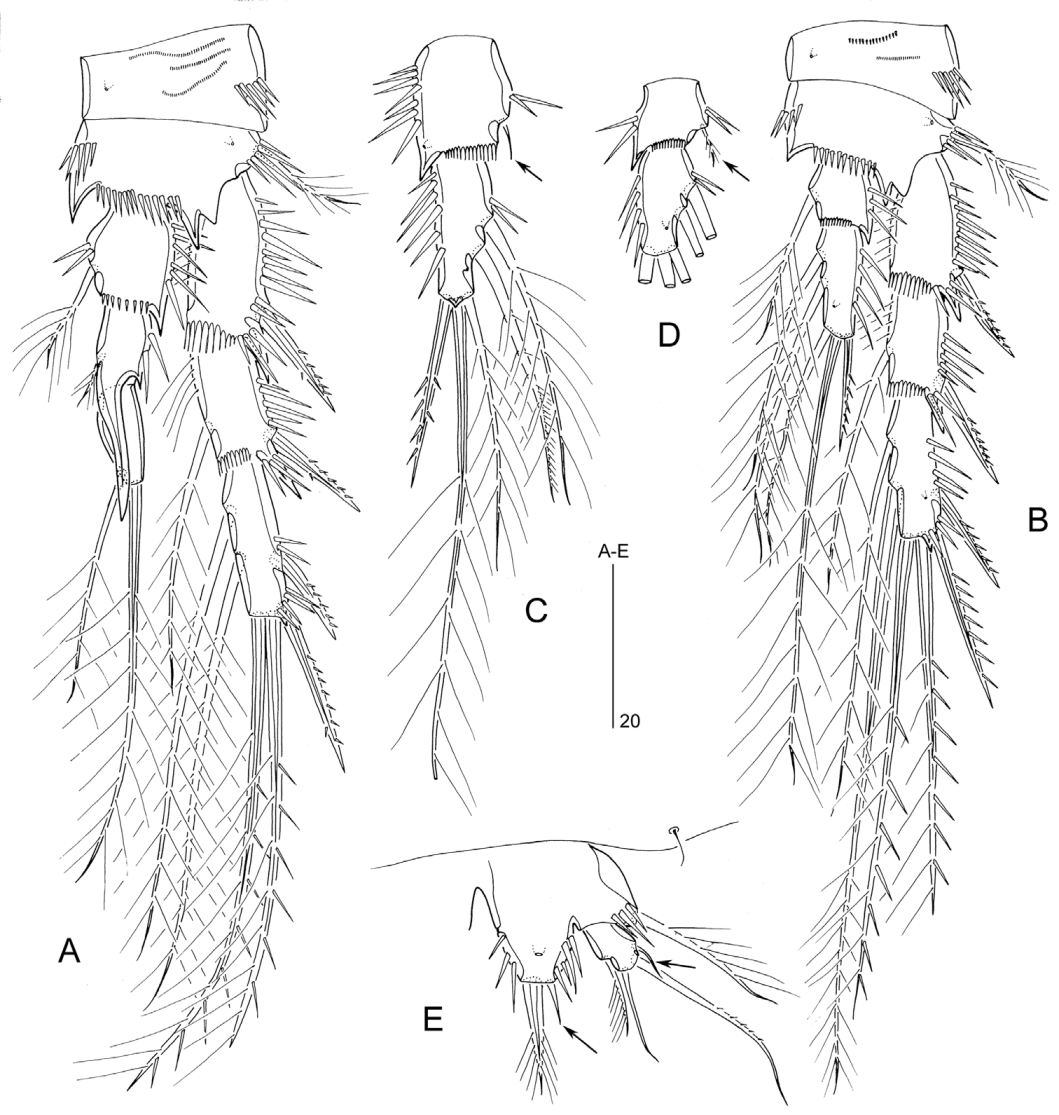
*Mesopsyllus spiniferus* sp. n.: **A** P3 ♂, anterior **B** P4 ♂, anterior **C** P3 endopod ♀, anterior (vestigial seta on enp-1 indicated by arrow) **D** P4 endopod ♀ (reduced seta on enp-1 indicated by arrow; setae on enp-2 not drawn at full length), anterior **E** P5 ♂, anterior (minute setae on exopod and endopodal lobe indicated by arrow) **A–B, E** based on holotype (NHMUK reg. no. 2013.1045) **C–D** based on paratype (NHMUK reg. no. 2013.1046).

P3 (Fig. 10A). Coxa, basis and exopod as in *M. dimorphus*. Inner seta of enp-1 much shorter than in *M. dimorphus*.

P4 (Fig. 10B). Coxa as in *M. dimorphus*. Basis with a spinous process at inner distal corner and between insertion sites of exopod and endopod. Distal inner seta of exp-3 thicker than proximal one. Inner seta of enp-1 much shorter than in *M. dimorphus*, only reaching distal margin of enp-2; enp-2 longer than in *M. dimorphus*, with two (instead of one) inner setae.

Seta and spine formula of P1–P4 as follows:

**Table:** 

**Thoracopod**	**Exopod**	**Endopod**
P1	0.1.022	1.1.111
P2	0.1.122	1.221
P3	0.1.222	1.1+apo.020 (1.221)
P4	0.1.222	1.221

Formulae in parentheses denote female condition.

P5 (Fig. 10E). Number of armature elements as in *M. dimorphus*. Endopodal lobe slightly wider and inner spine more spiniform and shorter than in *M. dimorphus*. Exopodal setae 1–2 relatively shorter compared to seta 3.

####### Description of female.

Body length 340–350 µm (n = 2, mean = 345 µm). Body covered with pattern of minute pimples (not figured). Sexual dimorphism in antennule, P3–P5, and urosomal segmentation and ornamentation.

Antennule, P5, and urosomal segmentation and ornamentation as in *M. dimorphus*.

P3 (Fig. 10C). Coxa, basis and exopod as in ♂. Enp-1 with minute inner seta and spinous process at outer distal corner; enp-2 with two inner setae.

P4 (Fig. 10D). Coxa, basis and exopod as in ♂. Enp-1 with minute, plumose inner seta (indicated by arrow).

####### Variability.

Both female specimens display right-left asymmetrical setal formulae on one pair of swimming legs. In the first specimen P3 enp-2 displays only one inner seta on one side and two on the other; in the second specimen P4 exp-3 exhibits one inner seta on one side but two on the other. The male holotype is aberrant in leg 1 with one side represented by a single segment with two distal setae and one outer spine (compare typical condition observed in dissected ♀ paratype: Fig. 9D).

####### Etymology.

The species name alludes to the two spiniform elements on segment 7 of the male antennule.

## Discussion

### Species differentiation and validity of *Vibriopsyllus* Kornev & Chertoprud, 2008

Por (1960) proposed the monotypic genus *Mesopsyllus* for a new species, *M. atargatis*, based on four females collected from muddy substrates at 51–82 m depth off the Romanian Black Sea coast. The species has only been recorded twice since its original description. Marinov and Apostolov (1981) recorded three females at 15 m depth in the Gulf of Piran (Slovenia) in the northern Adriatic. Bodin and Le Guellec (1992) found *M. atargatis* at 8.7–11 m depth in the Bay of Saint-Brieux, northern Brittany (France). Por’s (1960) description contains deficiencies and some of them, such as the doubtful armature pattern on the P2 endopod (cf. Table 2), have been pointed out by Lang (1965). *Mesopsyllus* females typically display one and two enlarged spinulose spines on antennulary segments 2–3, respectively (Fig. 3D); however, Por (1960) shows a divergent (and probably incorrect) pattern in *M. atargatis*, with segments 2–4 each bearing a single spine. His claim that the antennary exopod is absent is almost certainly false (the antenna was not illustrated) since this condition is not found in any of the other four congeners, all of which display a 1-segmented bisetose or trisetose exopod. Note that the antennary exopod of *M. atargatis* was therefore probably incorrectly scored as completely absent in Wells’ (2007) tabular key to the Canthocamptidae (his codon KG0; p. 203). According to Por’s (1960) text the armature formula for the distal exopodal segment of P4 is 223 but this is contradicted by his illustration which shows only one inner seta; this error was adopted by Por (1964a: 116) but subsequently corrected by Por (1964b: 252). *Mesopsyllus atargatis* is the only member of the genus that was described as having five elements on the endopodal lobe of the female leg 5. Morphological comparison with other species (all of which possess four elements) lends support to the assumption that the innermost element in reality represents one of the spinules typically found in this position (cf. Fig. 2D). His statement that the anal operculum is fringed with fine spinules is based on an observational error; the alleged spinular ornamentation in reality refers to the underlying incised frill bordering the anal opening.

Por (1964a) transferred *M. atargatis* to a more inclusive genus, *Hemimesochra*, effectively rendering *Mesopsyllus* a junior subjective synonym of the latter. Lang (1965) dismissed Por’s heterogeneous concept of *Hemimesochra* and restricted the genus to its type species *H. clavularis*, resurrected the monotypic *Mesopsyllus*, and transferred the third species, *H. derketo*, to a new genus *Poria*. The new replacement name *Hanikraia* was substituted for *Poria*, the latter being preoccupied by a genus of Coleoptera (Huys 2009). Wells (1965) described *Hemimesochra secunda* based on one female and one male collected at 101 m depth in Loch Nevis, a sea loch on the west coast of Scotland where it co-occurs with *H. clavularis*; the species has not been recorded again. Although no factual justification for its generic assignment was given it appears that Wells (1965) based his course of action primarily on the 2-segmented condition of the P1 endopod, a character shared with the type species *H. clavularis*. Huys and Thistle (1989) reviewed the genus *Hemimesochra* and transferred *H. secunda* to *Mesopsyllus* based on similarities with the type species in the morphology of the rostrum, female antennule and legs 2–4. Re-examination of the types (NHMUK reg. nos 1965.3.26.10–11) not only confirmed the 2-segmented P1 endopod (3-segmented in all other *Mesopsyllus* species) but also revealed minor errors in the original description of legs 1–2 (Table 2). *Mesopsyllus atargatis* and *M. secundus* differ from their congeners by the setulose anterior margin of the rostrum (*vs* naked), the presence of only one inner seta on P2 enp-2 (*vs* two) and of three outer spines on P4 exp-3 (*vs* two). They can be differentiated from each other by the segmentation of the P1 endopod, the number of inner setae on P4 exp-3, and the number of elements on the exopod of leg 5 in the female.

**Table 2.** Antennulary characters (# = number of segments; ae = segment on which proximal aesthetasc is located; sp = presence/absence of enlarged spinulose spines on segments 2–3) and P1–P5 armature formulae of species of *Mesopsyllus* Por, 1960 (*M.*) and related genera (*Ba.* = *Bathycamptus* Huys & Thistle, 1989; *Bo.* = *Boreolimella* Huys & Thistle, 1989; *Ha.* = *Hanikraia* Huys, 2009; *He.* = *Hemimesochra* Sars, 1920; *Ps.* = *Psammocamptus* Mielke, 1975; *Pu.* = *Pusillargillus* Huys & Thistle, 1989; *S.* = *Sympodella* gen. n.; ? = condition unknown).

	A1 ♀			P1		P2		P3		P4		P5 ♀			P5 ♂	
	#	ae	sp	exp	enp	exp	enp	exp	enp	exp	enp	rami	exp	benp	exp	benp
*M. atargatis*	6	4	+	0.1.022^a^	1.1.111	0.1.123	1.121^b^	0.1.223	1.121	0.1.123^c^	1.121	free	3	4^d^	?	?
*M. curvisetus*	6	4	+	0.1.022	1.1.111^e^	0.1.123	1?.221^f^	0.1.223	1.221	0.1.222	1.121	free	4	4	5	2
*M. dimorphus*	6	4	+	0.1.022	1.1.111	0.1.122	1.221	0.1.222	1.121	0.1.222	1.121	free	3	4	4	2
*M. secundus*	6	4	+	0.1.022^g^	1.111^h^	0.1.123^i^	1.121	0.1.223	1.121	0.1.223	1.121	free	5	4	4	2
*M. spiniferus*	6	4	+	0.1.022	1.1.111	0.1.122	1.221	0.1.222	1.221	0.1.222	1.221	free	3	4	4	2
*Ba. minutus*	?	?	+	0.1.022	1.1.111	0.1.122	1.121	0.1.222	?	0.1.222	?	?	?	?	5	2
*Ba. eckmani*	7	4	+	0.1.022	1.1.111	0.1.122	1.121	0.1.223	1.121	0.1.223	1.121	fused	4	4	5	2
*Ps. axi*	(7)^j^	4	+	0.1.022^k^	1.1.111^k^	0.0.022	(1).111^l^	0.0.122	(1).121^l^	0.(1).122^l^	1.121	fused	3	4	4	2
*Ps. galapagoensis*	7	4	+	0.1.022	1.1.111	0.1.122	1.111	1.1.122	1.121	0.1.122	1.121	fused	3	4	4	2
*He. clavularis*	5	3	+	0.0.022^m^	1.111	0.1.123	1.221	0.1.223	1.321	unknown^n^	unknown^n^	fused^o^	4	4	?	?
*Bo. dubia*	5^p^	3	+	0.1.022^q^	1.211^q^	0.1.122	1.121	0.1.122	1.121	0.1.122	1.121	fused	4	4	?	?
*Bo. nympha*	5	3	+	0.1.022	1.211^r^	0.1.122	1.221	0.1.222	1.221	0.1.222	1.221	fused	4	4	?	?
*Ha. derketo*	6^s^	3	–	0.0.022^m^	1.1.111	0.1.123^t^	1.1.111	0.1.223^t^	1.1.121	0.1.223	1.221	free	4	4	?	?
*Pu. nixe*	5	3	–	0.1.022	1.211	0.1.122	1.221	0.1.222	1.221	0.1.222	1.221	free	5	4	5	2
*S. galapagoensis*	6	3	–	0.1.022^m^	1.1.111	0.1.122	1.111	0.1.222	1.121	0.1.222	1.121	fused	5	4	6	2

^a^ Por erroneously tabulates the formula as 0.1.121 (1964a: 116) or 0.1.120 (1964b: 252).

^b^ According to Por’s (1960, 1964a, 1964b) illustrations and text the formula appears to be 0.111; the notches illustrated along the inner margin of both endopodal segments were interpreted by Lang (1965: 423) as insertion sites of setae which had been dislodged prior or during dissection, suggesting that the correct armature formula is 1.121 which is adopted here.

^c^ Por (1960) illustrates only one inner seta on exp-3 but lists the setal formula as 223; this error was adopted by Por (1964a: 116) but subsequently corrected by Por (1964b: 252).

^d^ According to Por’s (1960) text the endopodal lobe bears five elements; comparison with other species lends support to the assumption that the innermost element in reality represents one of the spinules typically found in this position.

^e^ Kornev and Chertoprud (2008) give the setal formula as 1.1.220, erroneously interpreting the long proximal spinule along the inner margin as an additional seta.

^f^ It is questionable whether Kornev and Chertoprud’s (2008) setal formula (0.221) is correct. Their illustration of leg 2 (fig. 5.123Б) shows a setule-like element on enp-1 in exactly the same position as the vestigial inner seta in *M. dimorphus* (Fig. 5C) and *M. spiniferus* (Fig. 9C), suggesting that the real setal formula is 1.221 as in the Chinese species.

^g^ Wells (1965) interprets the armature pattern of exp-3 as 121.

^h^ Although Wells (1965: Text-fig. 56) does not illustrate an inner seta on enp-1, its presence (as indicated by his setal formula 1.121) was confirmed by examination of the holotype (NHMUK reg. no. 1965.3.26.10); confirmation of the armature pattern on enp-2 is more problematic due to the bad preservation of the type slides, however, it appears that the short proximal outer element figured by Wells is in reality a spinule and that the distal formula is 111 as in other species of the genus; the outer distal element is spiniform rather than setiform and only half the size of that illustrated in the original description.

^i^ Wells (1965: Text-Fig. 57) gives the correct setal formula for exp-3 but the slender seta figured at the inner distal corner is distinctly longer and originates from the proximal half of the inner margin.

^j^ Mielke (1975) described the antennule as 6-segmented; his re-examination (Mielke 1997: 187) revealed the distal segment to be weakly subdivided.

^k^ Contrary to Mielke’s (1975) original description P1 exp-2 and enp-1–3 have a minute inner seta.

^l^ The rudimentary setules indicated as (1) in Mielke’s (1975) armature formula are genuine armature elements.

^m^ Mielke’s (1997: fig. 11A) description of *Carolinicola galapagoensis* showed that the inner seta on exp-2 can attain vestigial proportions. The absence of this element in *Ha. derketo* and *He. clavularis* therefore requires confirmation.

^n^ Sars’ (1920) description was based on two ♀♀ from Risør, southern Norway and illustrated only P1–P3 without giving any information about the armature pattern of leg 4. The only other record of the species is that by Wells (1965) who obtained three ♀♀ from Loch Nevis but refrained from making additional observations. Lang (1948: 1249) listed the formula for leg 4 as 0.1.223 for the exopod and 1.221 for the endopod. Since he did not collect material of the species nor re-examined the type material the armature pattern is probably based on Sars’s (1920) statement that the legs are “… of a structure similar to that in *Mesochra*” and should therefore be considered unconfirmed. Unfortunately this unverified fact has been perpetuated in the literature (Por 1964a, 1964b, Wells 1965, Coull 1973a).

^o^ According to Sars (1920) the exopod is discrete but Por (1964b: 254) claims that it is fused to the baseoendopod.

^p^ Wells (1965: Text-Fig. 93) shows a 6-segmented condition but claims in the text that the antennule is 5-segmented; the proximal “segment” represents a pedestal with which the antennule articulates.

^q^ There is controversy over the correct armature of leg 1 since there is a discrepancy between Wells’ (1965) text and illustration (his fig. 96 shows no inner seta on exp-2 and four elements on enp-2) (Becker 1972, Huys and Thistle 1989). Re-examination of the holotype (NHMUK reg. no. 1965.3.26.15) confirmed the presence of the inner seta on exp-2 and one inner and three apical elements on enp-2.

^r^ According to Por’s (1964b) text and illustration (Fig. X-111) the armature formula of the distal segment is 120. The inner seta on this compound segment is derived from the ancestral enp-2 segment while the two distal elements are derived from ancestral enp-3; it is assumed here that the short inner apical seta on the latter segment was overlooked by Por (cf. *M. spiniferus*, fig. 9D for ancestral 1.1.111 condition).

^s^ Por (1964a: Plate 21, fig. 243) shows a 7-segmented condition but states in the text that the antennule is 6-segmented with the proximal aesthetasc arising from the fourth segment; the armature of the proximal section of the antennule shows that the first unarmed “segment” is in reality the pedestal.

^t^ According to Por’s (1964a: Plate 21, figs 246–247) illustrations the inner seta on P2–P3 exp-2 is absent but the notches along the inner margin of both segments suggest that the setae were dislodged prior or during dissection; Por’s (1964a: 116) setal formulae indicate that the inner setae are present.

Kornev and Chertoprud (2008) described a new genus and species, *Vibriopsyllus curviseta* Kornev & Chertoprud, 2008, from shell gravel at 10 m depth in Rugozorskaya, an inlet of the Kandalaksha Gulf in the White Sea. The species was recently recorded from silty sand at 39 m depth in the Kara Sea (Garlitska and Azovsky 2016). Kornev and Chertoprud (2008) assigned *V. curviseta* to the Hemimesochrinae and considered it closest to *Hemimesochra clavularis* because of the morphological similarities in the antennule (6-segmented in ♀; although in *H. clavularis* it is only 5-segmented!), antenna (“conspicuous” allobasis with 1-segmented trisetose exopod; but note, however, that the antenna in *H. clavularis* has a genuine basis), maxillule (unisetose exopod, bisetose endopod), maxilla (syncoxa with two endites) and the female P5 (exopod small, with four setae; baseoendopod with four setae, seta III being very long). Despite these similarities Kornev and Chertoprud (2008) refrained from including their new species in *Hemimesochra* and considered the proposal of *Vibriopsyllus* warranted based on three differences, i.e. (a) P1 endopod 3-segmented (*vs* 2-segmented in *Hemimesochra*), (b) caudal ramus shape, and (c) P3 enp-2 with six elements (*vs* seven in *Hemimesochra*). Note that the last character must be an inadvertent slip of the pen since *V. curviseta* has in reality five while *H. clavularis* displays only six elements on this segment (cf. Sars, 1920: Plate XLV; Kornev and Chertoprud 2008: fig. 5.123B). Kornev and Chertoprud (2008) regarded the distinctive morphology of the female leg 5 as a synapomorphy supporting the sistergroup relationship between both genera. Presumably this statement referred to the armature rather than the segmentation since re-examination by Por (1964b: 254) revealed that the baseoendopod and exopod are fused in *H. clavularis* while they remain separate in *V. curviseta*. Although Kornev and Chertoprud (2008) cited Huys and Thistle’s (1989) revision of *Hemimesochra* and related genera they surprisingly did not compare *V. curviseta* with other members of the Hemimesochrinae. Within the latter, a lineage consisting of the genera *Mesopsyllus*, *Psammocamptus* and *Bathycamptus* shares with *V. curviseta* two antennulary characters, i.e. the position of the proximal aesthetasc (on segment 4 rather than segment 3) and the spinulose spine pattern (one on segment 2, two on segment 3 and one on the apical segment) (Table 2), the reduction of the inner seta on P1 enp-1, and the elongate caudal rami. *Psammocamptus* and *Bathycamptus* are closely related to each other since both display the same sexual dimorphism on leg 4 endopod (the presence of an additional inner seta on enp-2 in the ♂); based on this synapomorphy and the fused condition of leg 5 in both sexes the two genera can be considered sister taxa. Members of the genus *Mesopsyllus* all exhibit a 6-segmented antennule in the female, an apomorphic condition derived by failure of separation of the two apicalmost segments that is also shared by *V. curviseta*; see Huys and Thistle (1989: fig. 3A) and Mielke (1997: fig. 15A) for the ancestral 7-segmented condition in *Bathycamptus eckmani* Huys & Thistle, 1898 and *Psammocamptus galapagoensis* Mielke, 1997, respectively. Both *Hanikraia derketo* (Por, 1964a) and *Carolinicola galapagoensis* Mielke, 1997 also display a 6-segmented antennule, however, this condition is not homologous to the *Mesopsyllus* segmentation pattern since it originated from failure of separation of segments 3 and 4 (as indicated by the armature pattern and the position of the aesthetasc). Comparison between the type species of *Vibriopsyllus* and the four known species of *Mesopsyllus* (Table 2) shows that there are no grounds for maintaining the former as a distinct genus and hence it should be relegated to a junior subjective synonym of the latter. *Vibriopsyllus curviseta* is here formally transferred to *Mesopsyllus* as *M. curvisetus* (Kornev & Chertoprud, 2008), comb. n. The species is characterized by the presence of four elements on the female P5 exopod, and five elements on the male P5 exopod, a condition so far unique in the genus (Table 2).

The two Chinese species described herein, *M. dimorphus* and *M. spiniferus*, differ from their congeners by the presence of two instead of three outer spines on P2–P3 exp-3. They can be differentiated from each other by the following combination of characters: (1) number of inner setae on P3–P4 enp-2 (one in *M. dimorphus*, two in *M. spiniferus*); (2) anterior margin of antennulary segment 7 of male (with two spiniform elements in *M. spiniferus*; with three conical elements in *M. dimorphus*); (3) ornamentation of male urosome (second abdominal somite with paired dorsal spinular patches in *M. spiniferus*; absent in *M. dimorphus*); (4) presence/absence of sexual dimorphism on P2 endopod, P3–P4 exp-3 (present in *M. dimorphus*; absent in *M. spiniferus*); and (5) differences in length of setae on male P5.

The genus *Mesopsyllus* is so far restricted to the Northern Hemisphere. Soyer (1971) listed an as yet undescribed species as *Hemimesochra* sp. from Banyuls-sur-Mer, France. Baguley (2004) recorded eight undescribed species of *Mesopsyllus* from the deep sea in the Northern Gulf of Mexico. George (2013) listed an unidentified species from the Sedlo Seamount in the North Atlantic. An as yet undescribed *Mesopsyllus* species, possibly conspecific with *M. secundus*, was collected from a muddy substrate at 11 m depth in Loch Creran, Argyll in Scotland (E. Ólafsson, pers. commn). Grego et al. (2014) recently recorded an unnamed species of *Vibriopsyllus* during a field experiment on a silty sand bottom at 24 m depth in the northern Adriatic Sea. Undescribed species of the closely related genera *Psammocamptus* and *Bathycamptus* have been reported from the Gulf of Mexico (Baguley 2004, Brooks et al. 2009, Plum et al. 2015), the Porcupine Seabight (Gheerardyn et al. 2009), the Seine Seamount (Büntzow in George 2013), French Polynesia (Villiers and Bodiou 1996), Svalbard (Kotwicki 2002), and Kuwait (R. Huys, unpublished data).

### Key to species of *Mesopsyllus*

Caution must be exercised while attempting to identify species since some original descriptions contain inaccuracies and anomalous setation patterns in legs 1–5 are known to exist in some species so that observations based on a single specimen may not always reveal the usual (typical) condition. Members of the genus *Mesopsyllus* are typically small to very small and most original descriptions were based on very few specimens (Table 3). The swimming leg armature formulae of all species – with reinterpretations where required – are tabulated in Table 2. The key below is applicable to both sexes.

**Table 3.** Body length (in μm) and number of specimens (#) used in original descriptions of *Mesopsyllus* species.

Species	♀ (μm)	♂ (μm)	# ♀♀	# ♂♂
*M. atargatis*	470	?	4	?
*M. secundus*	400	340	1	1
*M. dimorphus*	240–330	220–280	11	3
*M. spiniferus*	340–350	280–320	2	2
*M. curvisetus*	405	not given	1	1

**Table:** 

1	Rostrum with setulose anterior margin; P2 enp-2 with one inner seta; P4 exp-3 with three outer spines	**2**
–	Rostrum with smooth anterior margin; P2 enp-2 with two inner setae; P4 exp-3 with two outer spines	**3**
2	P1 endopod 3-segmented; P4 exp-3 with one inner seta	***M. atargatis* Por, 1960**
–	P1 endopod 2-segmented; P4 exp-3 with two inner setae	***M. secundus* (Wells, 1965)**
3	P2–P3 exp-3 with two outer spines; P5 ♀ exopod with three elements; P5 ♂ exopod with four elements	**4**
–	P2–P3 exp-3 with three outer spines; P5 ♀ exopod with four elements; P5 ♂ exopod with five elements	***M. curvisetus* (Kornev & Chertoprud, 2008), comb. n.**
4	P3–P4 enp-2 ♀ with one inner seta; P2 endopod and P3–P4 exp-3 displaying sexual dimorphism as illustrated in Fig. 5A–B and Fig. 6A, B, D, F, respectively; second abdominal somite without paired dorsal spinular patches in ♂	***M. dimorphus* sp. n.**
–	P3–P4 enp-2 ♀ with two inner setae; P2 endopod and P3–P4 exp-3 without such sexual dimorphism; second abdominal somite with paired dorsal spinular patches in ♂	***M. spiniferus* sp. n.**

### Taxonomic position of *Carolinicola* Huys & Thistle, 1989 and proposal of *Sympodella* gen. n.

Coull (1973a) described *Hemimesochra trisetosa* based exclusively on females obtained from deep sea sediments off North Carolina. Huys and Thistle (1989) pointed out that the species was radically different from the species included in *Hemimesochra* at that time and established the genus *Carolinicola* to accommodate it. Mielke (1997) described both sexes of a second species, *C. galapagoensis*, from a sandy beach in the Galápagos archipelago, which he provisionally assigned to the genus. Additional, as yet undescribed, species have been reported from the Straits of Magellan and the Beagle Channel (George 1999, 2005), the abyssal plain of the Kuril Trench (Kitahashi et al. 2013) and from a marine cave near Marseille, France (Janssen et al. 2013).

Based on irreconcilable differences in the morphology of the rostrum, antenna, mandible and caudal rami, Huys and Thistle (1989) removed *Carolinicola trisetosa* (Coull, 1973a) from the Hemimesochrinae (Canthocamptidae) and placed it as an “advanced member” in the ”Paranannopidae” (= Danielsseniidae; see Huys (2009: 11) for a discussion on the availability of these family-group names). Willen (2000) likewise listed *Carolinicola* as a member of the subfamily Paranannopinae (= Danielsseniinae) in the Pseudotachidiidae, a view that was adopted by Wells (2007). However, the discovery of the male of *C. galapagoensis* led Mielke (1997) to suggest that both species of *Carolinicola* should remain in the same family as the former *Hemimesochra* species. Kim et al. (2011) endorsed Mielke’s (1997) view that *Carolinicola* has canthocamptid affinities (e.g., sexual dimorphism of P3 endopod), showing similarities with *Boreolimella* and *Poria* (= *Hanikraia*), and consequently removed the genus from the Danielsseniinae to the Hemimesochrinae. Unfortunately, Kim et al. (2011) neglected to address the heterogeneity of the genus. While the morphology of *C. galapagoensis* lends support to their course of action, the characters of the type species, *C. trisetosa*, clearly indicate that the latter (and – by inference – the genus *Carolinicola*) should remain in the Danielsseniinae as initially advocated by Huys and Thistle (1989). Within the Hemimesochrinae *C. galapagoensis* belongs to a lineage that is characterised by (1) proximal aesthetasc positioned on third antennulary segment due to failure of separation of ancestral segments 3–4, and (2) inner seta of P1 enp-1 long, pectinate and recurved both dorsally and backwardly. This combination of characters is expressed in members of the genera *Hemimesochra* (the presence of the posteriorly directed seta in *H. clavularis* was confirmed by Por (1964b: 254)), *Boreolimella*, *Hanikraia* and *Pusillargillus*. Both *Hanikraia derketo* and *Pusillargillus nixe* (Por, 1964b) share with *C. galapagoensis* the absence of enlarged spinulose spines on the antennule (typically one on second and apical segments, two on third segment) and the short caudal rami. *Hanikraia derketo* displays the most plesiomorphic swimming leg segmentation and armature, having 3-segmented endopods on legs 1–3 and three outer spines on P2–P4 exp-3 (Table 2). Both *P. nixe* and *C. galapagoensis* display the 2-segmented condition in the endopods of legs 1–4 and have only two outer spines on P2–P4 exp-3; based on these synapomorphies these species cannot be placed in *Hanikraia*. *Pusillargillus nixe* differs from *C. galapagoensis* in the segmentation of the P1 endopod, the presence of an enlarged outer spine on P1 exp-3, the unipinnate nature (with very long pinnules in distal half) of the outer exopodal spines on legs 2–4 and the separation of leg 5 exopod and baseoendopod in both sexes. *Carolinicola galapagoensis* has retained the 3-segmented condition of the P1 endopod but displays a more reduced armature on the endopods of legs 2–4 (Table 2). Based on this combination of mutually exclusive characters states, *C. galapagoensis* and *P. nixe* cannot be considered congeneric and consequently the former is here fixed as the type species of a new genus, *Sympodella* gen. n. whose diagnosis is given below.

### Family Canthocamptidae Brady, 1880

#### Subfamily Hemimesochrinae Por, 1986

##### Genus Sympodella gen. n.

Taxon classificationAnimaliaHarpacticoidaCanthocamptidae

http://zoobank.org/3D8C3AAA-42DC-4D11-AE0F-0EB5F870F134

###### Diagnosis.

Rostrum defined at base; triangular. Antennule 6-segmented in ♀, with aesthetasc on segments 3 and 6; 9-segmented, haplocer with geniculation between segments 7 and 8 in ♂; without enlarged modified spines in both sexes. Antenna with two abexopodal setae on allobasis; exopod 1-segmented, with three setae. Mandibular palp with elongate basis (with one seta), 1-segmented endopod (with four setae) and vestigial unisetose exopod. Maxillule with rami incorporated into basis. Maxilla with two endites on syncoxa; endopod fused to allobasis. Maxilliped with well developed seta on syncoxa. Swimming legs of ♀ with 3-segmented exopods and 3- (P1) or 2-segmented endopods (P2–P4); armature formulae as in Table 2. Inner seta of P1 enp-1 recurved backwardly and dorsally; outer spine of P1 exp-1 not enlarged; outer exopodal spines of P1–P4 bipinnate, without elongate pinnules in proximal half. Inner distal seta of P2 enp-2 and outer distal seta of P4 enp-2 longer in ♂. P3 endopod 3-segmented in ♂; enp-2 with inner seta and slender terminal apophysis; enp-3 with two apical setae. P5 with fused exopod and baseoendopod, forming weakly bilobate plate in both sexes; exopodal lobe with five and six elements in ♀ and ♂, respectively; endopodal lobe with four and two elements in ♀ and ♂, respectively. Sixth pair of legs asymmetrical in ♂, each with two tiny setae. Caudal ramus short, with six setae.

###### Type and only species.

*Carolinicola galapagoensis* Mielke, 1997 = *Sympodella galapagoensis* (Mielke, 1997) (by original designation).

###### Etymology.

The name is derived from the Greek *syn*, *sym*, meaning together, and *pous* (genitive *podos*), meaning foot, and refers to the fused condition of leg 5 in both sexes. Gender: feminine.

### A note on *Isthmiocaris* George & Schminke, 2003 and related genera

George and Schminke (2003) proposed the monotypic genus *Isthmiocaris* for a deepwater species, *I. longitelson* George & Schminke, 2003, from the Patagonian continental slope, and considered it most closely related to *Itunella* Brady, 1896 and *Bathycamptus*, primarily on account of the sexual dimorphism expressed in the endopods of P3–P4. Bruch et al. (2011) added a second species, *I. laurae* Bruch, Glatzel & Veit-Köhler, 2011 from the Angola Basin, which differed substantially from the type species in the segmentation and armature of the swimming legs (Table 4) and in the absence of the postcephalothoracic collar (or “isthmion” –– the primary diagnostic of the genus). While endorsing George and Schminke’s (2003) view on its relationships within the Canthocamptidae, they also recognized a close affinity (and potential synonymy) with the genus *Pyrocletodes* Coull, 1973b. The latter currently accommodates two deepwater species, *P. desuramus* Coull, 1973b from the deep sea off North Carolina and *P. coulli* Dinet, 1976 from the Angola Basin, both known exclusively from females (Coull 1973b, Dinet 1976) (Table 4). Coull (1973b) assigned *Pyrocletodes* to the Cletodidae but this course of action was disputed by Dinet (1976) who preferred to view its position as uncertain. For some inexplicable reason Por (1986) claimed that *Pyrocletodes* is a member of the Tetragonicipitidae, erroneously citing Dinet (1976) as the source for this familial assignment. This claim was considered a slip of the pen by several authors (Kunz 1994, Fiers 1995, Huys 1995). Huys et al. (1996) transferred the genus to the Canthocamptidae but gave no formal justification for this action while others continued to consider it as a *genus incertae sedis*, either in the Cletodidae (Wells 2007), the Podogennonta (Seifried 2003), the Syngnatharthra (Seifried and Schminke 2003) or the Harpacticoida (Bodin 1997). Based on the elongate, cylindrical habitus, caudal ramus shape, mouthpart morphology, and strongly reduced leg 5 there is no doubt that *Isthmiocaris* and *Pyrocletodes* are closely related and should be placed in the same family. Coincidently, these morphological attributes are also shared by another genus of doubtful affiliation, *Perucamptus*, which was established to accommodate a single species, *Hemimesochra rapiens*, from the Peru–Chile (Atacama) Trench (Becker 1979, Huys and Thistle 1989). All three genera could potentially be synonymous with *Pyrocletodes* taking priority over the other two. However, both *Pyrocletodes* and *Perucamptus* are known from females only and the sexual dimorphism expressed in the swimming legs is of primary importance in elucidating the relationships within the Canthocamptidae in general and the Hemimesochrinae in particular. For example, Huys and Kihara (2010) assigned *Metahuntemannia* Smirnov, 1946 and *Dahmsopottekina* Özdikmen, 2009 [note that Huys and Kihara (2010) cite the name *Pottekia* Huys, 2009, a new generic name intended to replace *Talpina* Dahms and Pottek, 1992 in Huys (2009) but which was subsequently withdrawn at the proof stage of that publication in favour of *Dahmsopottekina*] to the Hemimesochrinae based on the sexual dimorphism expressed on the P4 endopod (distal inner seta of the female modified into a serrate curved spine in the male; cf. *Pottekia pectinata* (Dahms & Pottek, 1992): Dahms and Pottek, 1992: fig. 35), a character indicating affinity with genera such as *Bathycamptus*, *Micropsammis* Mielke, 1975 and *Isthmiocaris*. In addition to the lack of information on the male, the difficulties in confirming the validity of *Perucamptus* are exacerbated by the fact that the type and only species, *P. rapiens* (Becker, 1979), may be based on a juvenile. Becker’s (1979) dorsal habitus drawing of the holotype suggests that he was dealing with a copepodid V stage, presumably a CV♀, since the purported genital double-somite is remarkably short for an adult female.

**Table 4.** Salient characters of members of *Pyrocletodes* Coull, 1973b, *Perucamptus* Huys & Thisle, 1989 and *Isthmiocaris* George & Schminke, 2003. [A1 ♀: number of segments and position of aesthetasc (ae); A2 exp = number of setae on antennary exopod; P3 enp: apo = apophysis; P5: b = outer basal setae, sp = spine(s); –– = absent].

Species	sex	A1 ♀	A2	P1		P2		P3		P4		P5
			exp	exp	enp	exp	enp	exp	enp	exp	enp	
*Pyrocletodes desuramus*	♀	5 (ae on 3)	2	0.1.022	1.020	0.1.122	––	0.1.122	––	0.1.122	––	b + 2
*Pyrocletodes coulli*	♀	5 (ae on 3)	3	0.0.021	1.020	0.1.122	––	0.1.122	––	0.1.122	––	b + 2
*Perucamptus rapiens*	?†	5 (ae on 3)	3	0.0.022	1.121	0.1.122	0.121	0.1.122	0.020	0.1.122	––	b+2 + 2
*Isthmiocaris longitelson*	♀	6 (ae on 4)	3	0.021	010	0.020	––	0.1.021	––	0.021	––	1
	♂		3	0.021	010	0.021	––	0.1.021	0.apo.020	0.0.021	0.021	b + 2 + sp
*Isthmiocaris laurae*	♀	6 (ae on 3)	3	0.0.022	0.011	0.0.022	020	0.0.022	020	0.0.022	010	b + 2
	♂		3	0.0.022	0.021	0.1.122	1.321	0.1.222	1.apo.020	0.1.222	0.221	b + 5 + 2sp
	CV♀	?	?	0.0.022	0.021	0.0.022	0.020	0.0.022	0.020	0.0.022	010	?
	CV♂	?	?	0.0.022	0.021	0.1.122	0.121	0.1.122	0.0.021	0.1.022	0.011	?

† Becker’s (1979) dorsal habitus drawing of the holotype suggests that he was dealing with a copepodid V stage, presumably a CV♀.

## Supplementary Material

XML Treatment for Mesopsyllus

XML Treatment for Mesopsyllus dimorphus

XML Treatment for Mesopsyllus spiniferus

XML Treatment for Sympodella
